# The Zygotic Division Regulator ZAR1 Plays a Negative Role in Defense Against *Botrytis cinerea* in *Arabidopsis*

**DOI:** 10.3389/fpls.2021.736560

**Published:** 2021-10-26

**Authors:** Lijuan Chen, Jiahui Xiao, Yuxiao Song, You Li, Jun Liu, Huiren Cai, Hong-Bin Wang, Bing Liu

**Affiliations:** ^1^Guangdong Provincial Key Laboratory of Plant Resources, School of Life Sciences, Sun Yat-sen University, Guangzhou, China; ^2^Institute of Medical Plant Physiology and Ecology, School of Pharmaceutical Sciences, Guangzhou University of Chinese Medicine, Guangzhou, China

**Keywords:** ZYGOTIC ARREST 1, CERK1, plant resistance, *Botrytis cinerea*, *Arabidopsis*

## Abstract

A phosphorylation/dephosphorylation cycle at tyrosine 428 of CHITIN ELICITOR RECEPTOR KINASE 1 (CERK1) plays an essential role in chitin triggered immunity in *Arabidopsis thaliana*. In this study, we used a differential peptide pull-down (PPD) assay to identify factors that could participate downstream of this cycle. We identified ZYGOTIC ARREST 1 (ZAR1) and showed that it interacts with CERK1 specifically when the tyrosine 428 (Y428) residue of CERK1 is dephosphorylated. ZAR1 was originally characterized as an integrator for calmodulin and G-protein signals to regulate zygotic division in *Arabidopsis*. Our current results established that ZAR1 also negatively contributed to defense against the fungus *Botrytis cinerea* and played a redundant role with its homolog ZAR2 in this process. The *zar1-3 zar2-1* double mutant exhibited stronger resistance to *B. cinerea* compared with *zar1-3* single mutant, *zar2-1* single mutant, and wild-type plants. Moreover, the inducible expression of numerous defense response genes upon *B. cinerea* infection was increased in the *zar1-3zar2-1* double mutant, consistent with a repressive role for ZAR proteins in the defense response. Therefore, our findings provided insight into the function of ZAR1 in multiple defenses and developmental regulation pathways.

## Introduction

Plants confront attacks from various pathogens and have developed immunity mechanisms to defend against pathogen infection. Cell-surface localized receptors termed pattern recognition receptors (PRRs) detect microbe-associated molecular patterns (MAMPs) and mediate signal transduction (Couto and Zipfel, 2016; Zipfel and Oldroyd, 2017). In *Arabidopsis thaliana*, the bacterial flagellin peptide (flg22), elongation factor Tu peptide (elf18), and fungal chitin are the best-characterized MAMPs (Boller and Felix, 2009). The cognate receptors for flg22, elf18, and chitin have been identified as flagellin-sensitive 2 (FLS2), EF-TU receptor (EFR), and LYSM-containing receptor-like kinase 5 (LYK5), respectively (Gomez-Gomez and Boller, 2000; Zipfel et al., 2006; Cao et al., 2014). The perception of MAMPs by PRRs activates downstream signal transduction networks, resulting in various immune responses including mitogen-activated protein kinase (MAPK) activation, the burst of reactive oxygen species (ROS), and inducible expression of defense genes (Couto and Zipfel, 2016; Gong et al., 2020).

In *Arabidopsis*, the chitin elicitor receptor kinase 1 (CERK1)/LysM-RLK1 is an indispensable receptor-like kinase (RLK) for chitin-triggered immunity signaling (Miya et al., 2007; Wan et al., 2008; Liu et al., 2012). Both heterodimerization between CERK1 and LYK5 and chitin-triggered homodimerization of CERK1 play essential roles in chitin signaling in *Arabidopsis* (Liu et al., 2012; Cao et al., 2014). Chitin-activated CERK1 phosphorylates downstream receptor-like cytoplasmic kinases (RLCKs) to regulate the chitin triggered MAPK activation and ROS burst (Zhang et al., 2010; Yamada et al., 2016; Liu et al., 2018). CERK1 activation is regulated by a phosphorylation/dephosphorylation cycle at the tyrosine 428 (Y428) residue to dynamically control immunity signaling (Liu et al., 2018). After chitin elicitation, CERK1 recruits the CERK1-interacting protein phosphatase 1 (CIPP1) to dephosphorylate Y428, which suppresses CERK1 signaling. CIPP1 subsequently dissociates from CERK1 harboring the dephosphorylated Y428 residue, allowing CERK1 to return to a standby state (Liu et al., 2018).

Candidate chitin-signaling regulators have been identified in *Arabidopsis* based on their ability to interact with CERK1 (Gong et al., 2020). For example, the RLK IOS1 can physically associate with CERK1 to facilitate chitin signaling (Yeh et al., 2016). Similarly, the RLK FERONIA can promote responses to chitin signal, whereas the RALF23 ligand-bound FERONIA plays the opposite role (Stegmann et al., 2017). In addition, *Arabidopsis* LIK1 is an interacting partner of CERK1 that plays a negative regulatory role in chitin-induced responses (Le et al., 2014). However, the factors involved in detecting and binding to the CERK1 motif containing the phosphorylated/dephosphorylated-Y428 residue remain largely unknown. In this study, we reported that the leucine-rich repeat RLK (LRR-RLK) ZYGOTIC ARREST 1 (ZAR1) could interact with CERK1 when it harbored the dephosphorylated Y428 residue and that ZAR1 negatively contributed to the defense against *Botrytis cinerea* in *Arabidopsis*.

## Materials and Methods

### Plant Materials and Growth Conditions

The seeds of *A. thaliana* used in this study, including the wild-type Columbia-0 (Col-0) ecotype, *zar1-4* mutant (SALK_143663), and *zar2-1* mutant (SALK-110111c) were obtained from the Nottingham *Arabidopsis* Stock Centre.^1^ The T-DNA insertion mutant *zar1-3* (SALK_021338)was kindly provided by Wei-Cai Yang from the Institute of Genetics and Developmental Biology, Chinese Academy of Sciences, Beijing China. The *cerk1* mutant was kindly provided by Gary Stacey. All plants were grown under 22°C, a 12-h/12-h light/dark cycle, and relative air humidity of 60–70%.

### Isolation of Protoplasts

Protoplast isolation from *Arabidopsis* seedlings was performed as previously described (Zhai et al., 2009) with modifications. Briefly, 14-day-old *Arabidopsis* seedlings were cut into strips and then incubated in the digestion solution containing.5 M mannitol, 10 mM MES-KOH (pH 5.7), 20 mM CaCl_2_, 40 mM KCl,1% Cellulase R-10 (Yakult Honsha Co., Ltd., Japan), and.3% Macerozyme R-10 (Yakult Honsha Co., Ltd., Japan) with gentle shaking for 3 h in darkness. The protoplasts were collected by centrifugation at 100 *g* for 7 min and washed twice with the W5 solution containing.1% glucose, 0.08% KCl, 0.9% NaCl, 1.84% CaCl_2_, and 2 mM MES-KOH (pH 5.7).

### Peptide Pull-Down Assay

For differential peptide pull-down (PPD) screening, total proteins derived from.5 g 10-day-old *Arabidopsis* seedlings were extracted with protein extraction buffer (10 mM HEPES (pH 7.5), 150 mM NaCl, 1 mM EDTA, 10% glycerol, 0.5%Triton X-100, 1 × piece protease inhibitor EDTA-free (Thermo, Fisher Scientific Inc., MA, United States), and 1 × piece phosphatase inhibitor Mini tablets (Thermo Fisher Scientific Inc., MA, United States), and incubated with 30 μl peptide^pY428^-beads or peptide^Y428^-beads for 4 h at 4°C with gentle shaking. Proteins not binding with beads were removed with extensive washing, and then the beads-binding proteins in each sample were then analyzed with sodium dodecyl sulfate-polyacrylamide gel electrophoresis (SDS–PAGE), followed by silver staining (Fast Silver Stain Kit, Beyotime, China). The differential protein bands between two samples were identified through mass spectrometry.

For pull-down verification of ZAR1-peptide^Y428^ interaction, 2 ml *Arabidopsis* protoplasts (∼10^6^ cells) were transfected with 200 μg plasmids DNA (*35S:ZAR1-HA*) and cultured in darkness. After 12 h of expression, the total proteins of protoplasts were extracted with 300 μl protein extraction buffer [10 mM HEPES (pH 7.5), 150 mM NaCl, 1 mM EDTA, 10% glycerol, 0.5% Triton X-100, 1 × piece protease inhibitor EDTA-free, and 1 × piece phosphatase inhibitor Mini tablets]. The total cell extracts were incubated with 30 μl peptide^pY428^-beads or peptide^Y428^-beads for 4 h at 4°C with gentle shaking. The beads-binding protein was detected by immunoblotting with HRP-conjugated anti-HA antibody.

### Co-immunoprecipitation Assay

**FIGURE 1:**
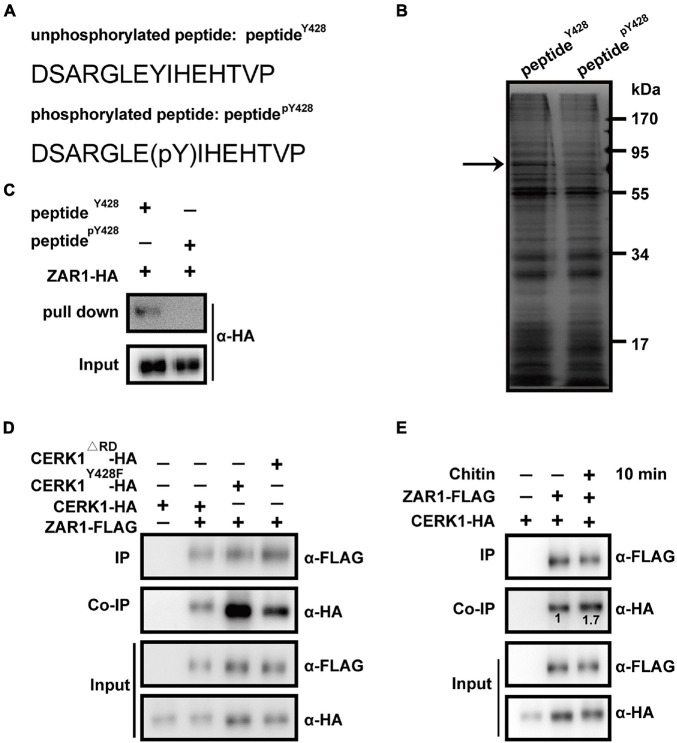
Zygotic arrest 1 (ZAR1) interacts with chitin elicitor receptor KINASE 1 (CERK1) depending on the dephosphorylation of Y428 residue and functions in defense against *Botrytis cinerea.* **(A)** Two “bait” peptides, peptide^pY428^ fragment “DSARGLE(pY)IHEHTVP” and peptide^Y428^ fragment “DSARGLEYIHEHTVP” was designed *via* the web-based *motif-x* program (http://motif-x.med.harvard.edu/). **(B)** Sodium dodecyl sulfate-polyacrylamide gel electrophoresis (SDS-PAGE) analysis of differential peptide-pulling down (PPD) proteins. After the PPD assay, the peptide^Y428^ beads-binding proteins and peptidep^Y428^ beads-binding proteins were analyzed with SDS-PAGE and followed by silver staining. The differential protein bands were observed. The differential band was illustrated with an arrow. **(C)** Pulling-down of ZAR1 protein with peptide^Y428^ beads. The HA-tagged ZAR1 protein expressed in *Arabidopsis* protoplasts was incubated with peptide^Y428^ beads, and the pulling-down proteins were detected by immunoblotting using an anti-HA antibody. Total protoplast proteins were used as input. The experiments were repeated three times with similar results. **(D)** Co-immunoprecipitation (co-IP) of ZAR1 and entire CERK1 protein. FLAG-tagged ZAR1 with HA-tagged CERK1^Y428*F*^, FLAG-tagged ZAR1 with CERK1^Δ^ ^RD^, or FLAG-tagged ZAR1 with wild type CERK1 was co-expressed in *Arabidopsis* protoplasts for 12 h, respectively. CERK1^Y428*F*^ and CERK1^Δ^ ^RD^ protein showed enhanced interaction with ZAR1 protein. The experiments were repeated three times with similar results. **(E)** Interaction between ZAR1 and CERK1 was influenced by chitin elicitation. HA-tagged CERK1 and FLAG-tagged ZAR1 were co-expressed in protoplasts for 12 h, then the co-IP was performed before chitin treatment or after 10 min of chitin treatment. Numbers indicate relative amounts of CERK1-HA co-immunoprecipitated with ZAR1-FLAG, measured using the ImageJ program. The experiments were repeated three times with similar results.

For co-immunoprecipitation (co-IP) assay in Figure 1D, 2 ml of protoplasts were transfected with 200 μg plasmids DNA, to co-express the ZAR1-FLAG protein with CERK1-HA, CERK1^ Y428*F*^-HA, or CERK1 ^△^^RD^-HA proteins, respectively. The protoplasts transfected with CERK1-HA plasmids only were used as the negative control. After protein expression for 12 h, the protoplasts were harvested and total proteins were extracted with 300 μl protein extraction buffer [10 mM HEPES (pH 7.5), 150 mM NaCl, 1 mM EDTA, 10% glycerol, 0.5% Triton X-100, 1 × piece protease inhibitor/EDTA-free, and 1 × piece phosphatase inhibitor Mini tablets]. The co-IP assay in *Arabidopsis* protoplasts was conducted according to a detailed protocol described previously (Cheng et al., 2015). Afterward, 10 μl of protein extracts of each sample was used as the input fraction, the remaining crude extracts of each sample were further incubated with 20 μl anti-FLAG M2 affinity gel (Sigma-Aldrich, United States) for 4 h at 4°C with gentle shaking. After washing three times with ice-cold extraction buffer, the gel-bound proteins were eluted by boiling with 30 μl 2 × SDS-PAGE loading buffer. The presence of ZAR1-FLAG and CERK1-HA in the eluate (IP fraction) and input fraction was analyzed by SDS-PAGE and immunoblotting with HRP-conjugated anti-HA or HRP-conjugated anti-FLAG antibodies.

For co-IP assay in Figure 1E, HA-tagged CERK1 and FLAG-tagged ZAR1 were co-expressed in protoplasts for 12 h. After being treated with 50 μg/ml chitin or not, the protoplasts were harvested, and total proteins were extracted according to the previous description. The protoplasts transfected with CERK1-HA plasmids were only used as the negative control.

### *Botrytis cinerea* Infection

*Botrytis cinerea* (CECT2100, Spanish Type Culture Collection, University de Valencia, Valencia, Spain) was cultured on PDA plates at 22°C for 10 days. The spores were washed, collected with potato dextrose broth (PDB), and diluted to 10^7^ spores/ml. For each infection, 5 μl droplet of spores was inoculated onto the surface of one leaf of the 4-week-old *Arabidopsis* plants. The leaves were detached from plants and cultivated in a high-humidity environment. After 48–72 h of inoculation, the lesion size was recorded and measured with Image J software.

For measurement of photosystem II (PSII) activity and cell death phenotype, the 4-week-old *Arabidopsis* plants were sprayed with a *B. cinerea* spore suspension and incubated under high humidity in darkness (Zhang et al., 2018).

### Analysis of Photosystem II Activity

The PSII activity was analyzed by measuring chlorophyll fluorescence. Chlorophyll fluorescence of *Arabidopsis* plants was measured using the MAXI version of the IMAGING-PAM M-Series chlorophyll fluorescence system (Heinz-Walz Instruments) according to the previous description (Lu et al., 2011; Zhang et al., 2018). The quantum efficiency of light-adapted leaves (ΦPSII) was calculated as (Fm′–F)/Fm′ according to the previous description (Lu et al., 2011).

### Trypan Blue Staining

Trypan blue solution contained 10 g phenol, 10 ml glycerol, 10 ml lactic acid, 10 ml water, and.02 g of trypan blue together (stock solution). The working solution was prepared by diluting the stock solution with ethanol (1:3 V/V). The leaves of 4-week-old *Arabidopsis* plants which were sprayed with *B. cinerea* spore suspension were boiled in staining solution at 95°C for 10 min. After cooling to room temperature, the leaves were de-stained in chloral hydrate solution (chloral hydrate:H_2_O:glycerol; 4:2:1 W/V/V) overnight. Images were captured under a Zeiss stereomicroscope (SteREO Lumar version 12, Germany).

### Mitogen-Activated Protein Kinase Assay

Ten-day-old Arabidopsis seedlings grown in the.5 × MS liquid medium were treated with 200 μg/ml chitin at different times before the seedlings were frozen in liquid nitrogen. Total proteins were extracted with the protein extraction buffer [10 mM HEPES (pH 7.5), 150 mM NaCl, 1 mM EDTA, 10% glycerol, 0.5% Triton X-100, 1 × piece protease inhibitor EDTA-free, and 1 × piece phosphatase inhibitor Mini tablets]. The protein lysates were resolved in a 10% SDS-PAGE gel and activated MAPKs were visualized by immunoblotting with anti-pERK antibodies (Cell Signaling Technology, United States).

### Oxidative Burst Assay

Reactive oxygen species measurement was performed as previously described (Zhang et al., 2007). In brief, rosette leaves from 6-week-old Arabidopsis plants were sliced into approximately 1 mm strip and incubated overnight in water in a 96-well culture plate. The next day, the water was replaced by a reaction solution containing.5 mM L-012 (Wako, Japan), 1 mg/ml horseradish peroxidase, and 200 μg/ml chitin (or water as the mock), and the chemiluminescence was measured in a time-course manner using a microplate reader (Varioskan LUX, Thermo Fisher Scientific Inc., United States or Tecan-Spark, Tecan, Switzerland). Each data point represents the average of six replicates.

### Transcriptome Sequencing and Data Analysis

Previous studies revealed a major transcriptional shift occurred around 20–28 h post-infection coinciding with the lag phase in *B. cinerea* growth (Windram et al., 2012). Therefore, 28 HPI was chosen as the time point of transcriptome sequencing in this study. Moreover, the *zar1-3 zar2-1* double mutant has shown stronger *B. cinerea* resistance compared with Col-0 in the early growth stage, which is suitable for the isolation of high-quality RNA. Then RNA-Seq analysis was performed with 12-day-old Col-0 and *zar1-3 zar2-1 Arabidopsis* seedlings sprayed with *B. cinerea* or PDB. After 28 h of treatment, the total RNA derived from leaves of each sample was purified for transcriptome sequencing by Beijing Genomics Institute (BGI, Shenzhen, China). Three independent biological replicates were being performed in each experiment group. Gene Ontology (GO) enrichment analysis was performed on a website^2^ based on a hypergeometric test on the proportion of GO categories between the differentially expressed genes (DEGs) and the whole genome; while the FDR of the test was <0.05. GO categories were considered enriched. The sum of all transcript per million (TPM) values is the same in all samples, such that a TPM value represents a relative expression level that is comparable between Col-0 and *zar1-3zar2-1* double mutant, or different treatment conditions.

Raw Illumina sequence reads were available in the Sequence Read Archive (SRA) of the NCBI under BioProject ID: PRJNA757130.^3^

### Quantitative RT-PCR

The 12-day-old *Arabidopsis* seedlings grown in the 1/2 MS medium were sprayed with *B. cinerea* or PDB for 28 h. Total RNA derived from leaves of each sample was extracted using a Plant RNA Kit (Magen, China) and 5 mg total RNA was used for cDNA synthesis using the PrimeScript RT reagent kit (Takara, Japan). qPCR was performed using SYBR Premix Ex Taq (Takara, Japan), and amplification was monitored in real-time on the LightCycler 480 (Roche). Reactions were performed in triplicate for each sample, and relative expression levels were normalized using the *UBQ5* gene. The primers used for qRT-qPCR are listed in Supplementary Table 2.

## Results

### ZAR1 Interacts With the CERK1 Motif Containing the Dephosphorylated Y428 Residue

To screen for candidate proteins interacting with the CERK1 motif, specifically with or without phosphorylation of the Y428 residue, we performed a differential PPD assay. A ‘‘bait’’ peptide fragment of CERK1 containing the Y428 residue was designed *via* the web-based *motif-x* program^4^ (Schwartz and Gygi, 2005; Chou and Schwartz, 2011). The peptide^pY428^ fragment “DSARGLE(pY)IHEHTVP” and the peptide^Y428^ fragment “DSARGLEYIHEHTVP” were synthesized and individually conjugated to agarose beads (Figure 1A). For PPD screening, total proteins derived from 10-day-old *Arabidopsis* seedlings were incubated with peptide^pY428^- or peptide^Y428^-conjugated beads. The peptide^pY428^- or peptide^Y428^-bound proteins were analyzed with SDS-PAGE and the differential protein bands between the samples were further analyzed using mass spectrometry (Figure 1B). Notably, the results indicated that ZAR1 (AT2G01210) could interact with peptide^Y428^, but not peptide^pY428^ (Figure 1C).

### ZAR1 Interacts With CERK1 Protein-Containing Dephosphorylated Y428

We investigated the interaction between ZAR1 and the entire CERK1 protein containing the dephosphorylated Y428 site *in vivo*. The Y428 phosphorylation is abolished in CERK1^Y428*F*^, in which the 428^*th*^ tyrosine residue of CERK1 is substituted with phenylalanine (F), and in a catalytically inactive CERK1 mutant (CERK1^Δ^ ^RD^; Liu et al., 2018). We evaluated the co-IP of FLAG-tagged ZAR1 protein with HA-tagged wild-type CERK1, CERK1^Y428*F*^, or CERK1^Δ^ ^RD^ in the *Arabidopsis* protoplasts. While ZAR1 showed weak interaction with the wild-type CERK1-HA protein, the ZAR1-CERK1 interaction was enhanced in the presence of the Y428F or ΔRD mutation in CERK1 protein (Figure 1D).

Notably, Y428 phosphorylation is a consequence of CERK1 autophosphorylation, and the Y428 phosphorylation level was dynamic during chitin signaling (Liu et al., 2018). We hypothesized that the interaction between ZAR1 and CEKR1 could be influenced by chitin elicitation. Indeed, ZAR1-CERK1 interaction was enhanced after 10 min of chitin treatment (Figure 1E), which was consistent with our earlier observation that phosphorylation of the Y428 residue gradually declined within 0–10 min upon chitin treatment (Liu et al., 2018).

The minor ZAR1-CERK1 interaction was also observed in the absence of chitin (Figures 1D,E). Most of the CERK1 proteins might be autophosphorylated at the Y428 residue in the standby state without chitin signal, while minor dephosphorylation of Y428 residue occurred on occasion, possibilities that need further investigation. Taken together, our differential PPD assay and co-IP data indicated that ZAR1 interacted with CERK1 protein harboring dephosphorylated Y428 residue.

### ZAR1 Negatively Contributes to Plant Defense Against *Botrytis cinerea* Independently of Chitin-Triggered Responses

ZAR1 encodes a receptor-like kinase and regulate the division of zygote in *Arabidopsis* (Yu et al., 2016). The homozygous *zar1-1*^–/–^ mutation (in ecotype Landsberg *erecta*) is embryo lethal, and the strong phenotype is most likely caused by the expression of the truncated ZAR1 protein, which functions in a dominant-negative manner. By contrast, plants harboring the null allele *zar1-2* (in ecotype Landsberg *erecta*) and *zar1-3* (in ecotype Col-0) are fertile, which may be due to the functional redundancy of ZAR1 homologs (Yu et al., 2016). To date, whether ZAR1 is involved in biological processes other than zygotic division regulation is still uncovered.

**FIGURE 2:**
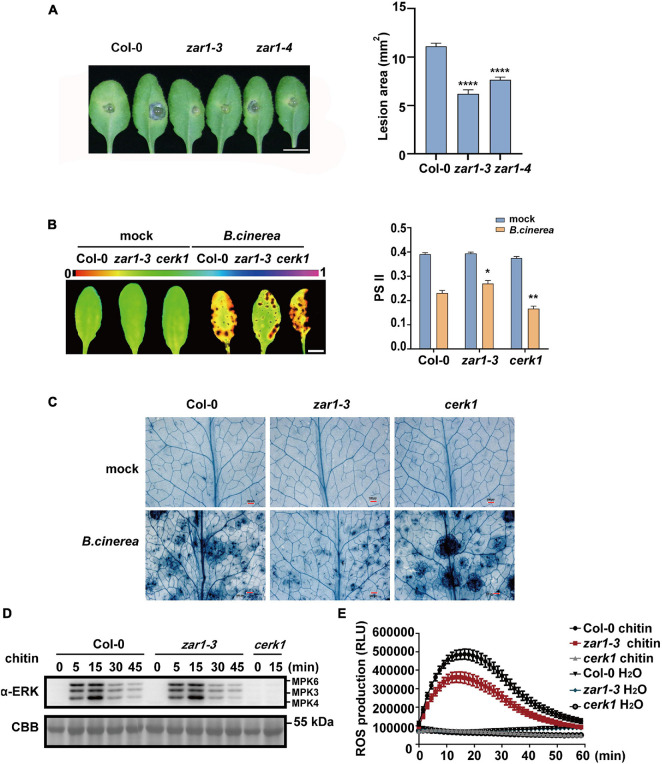
ZAR1 functions in the resistance to the fungal pathogen *Botrytis cinerea*. **(A)** ZAR1 functions in the resistance to the fungal pathogen *Botrytis cinerea*. Lesions on the leaves of *zar1-3*, *zar1-4* mutant, and Col-0 plant were observed at 48 h after inoculation with *B. cinerea*. Lesion sizes were analyzed. Error bars represent SEM from means of 16 leaves from each line. Asterisks indicate significant differences compared with Col-0 plant (****P < 0.0001, Student’s *t*-test). Scale bar = 1 cm. **(B)** Chlorophyll fluorescence imaging and quantification of ΦPSII was measured in *zar1-3* mutant, *cerk1* mutant, and Col-0 plant, at 36 h after *B. cinerea* spray. Error bars represent SEM of 16 leaves from each line. Asterisks indicate significant differences between each mutant and Col-0 plant (*P < 0.05, **P < 0.01, Student’s *t*-test). **(C)** Visualization of cell death in infected leaves at 36 h post-infection. Representative images of trypan blue (TPB) stained leaves are shown. Scale bar = 500 μm. **(D)** Chitin-triggered MAPK activation in *zar1-3* mutant, *cerk1* mutant, and wild type Col-0. 10-day-old seedlings were treated with 200 μg/mL chitin at the indicated time points. MAPK activation was detected with an anti-pERK antibody. *cerk1* mutant was included as a control. Equal loading is demonstrated by CBB (Coomassie brilliant blue) staining of Rubisco (below). The experiments were repeated three times, with similar results. **(E)** Leaves of the 4-week-old *zar1-3* mutant, *cerk1* mutant, and Col-0 plants were treated with 200 μg/ml chitin and incubated with luminol and horseradish peroxidase to detect ROS. Luminescence was recorded at different time points as indicated. Error bars represent SEM of data derived from replicate samples (*n* = 12). The chemiluminescence was measured in a time-course manner using a microplate reader (Varioskan LUX, Thermo Fisher Scientific Inc., MA, United States).

To explore whether ZAR1 is also involved in plant defense, we evaluated the sensitivity to fungal pathogen *Botrytis cinerea* of two *zar1* mutant lines in the Col-0 background, *zar1-3* (SALK_021338) and *zar1-4* (SALK_143663). After 48 h of *B. cinerea* infection, both *zar1-3* and *zar1-4* mutants showed smaller lesion sizes on leaves compared with those of Col-0, indicating the stronger resistance against *B*. *cinerea* in both *zar1-3* and *zar1-4* mutants (Figure 2A). We further characterized this phenotype by measuring photosystem II (PSII) activity and cell death in *zar1-3*, *cerk1*, and Col-0 plants that had been sprayed with *B. cinerea* spore suspension, as previously described (Zhang et al., 2018). The *zar1-3* mutant showed stronger resistance against *B. cinerea*, including smaller lesion sizes, higher PSII system activity, and less cell death compared with Col-0 plants (Figures 2B,C). By contrast, the *cerk1* mutant was more sensitive to *B. cinerea* compared with Col-0 plants (Figures 2B,C). These results imply that ZAR1 negatively contributes to defense against *B. cinerea* in *Arabidopsis*.

As chitin is the best-characterized MAMP of fungal pathogens, we evaluated chitin-triggered immunity responses, focusing on the early stage. We assessed mitogen-activated protein kinases (MAPK) activation and reactive oxygen species (ROS) burst in the *zar1-3* mutant upon chitin treatment. As the negative control, the *cerk1* mutant completely lost the chitin-induced MAPK activation and ROS burst, as previously observed (Figures 2D,E; Liu et al., 2018). However, there was no difference in MAPK activation between *zar1-3* and Col-0 plants (Figure 2D). In addition, the *zar1-3* mutant exhibited compromised ROS burst compared with Col-0 upon chitin treatment (Figure 2E). Thus, ZAR1 may play a minor role in the positive regulation of chitin signaling, although ZAR1 contributes negatively to resistance against *B. cinerea*. Taking these data together, we conclude that the enhanced resistance to *B. cinerea* in *zar1-3* mutant seems to be independent of early chitin-triggered responses such as MAPK activation and ROS burst.

### ZAR1 and ZAR2 Function Redundantly in Defense Against *Botrytis cinerea*

**FIGURE 3:**
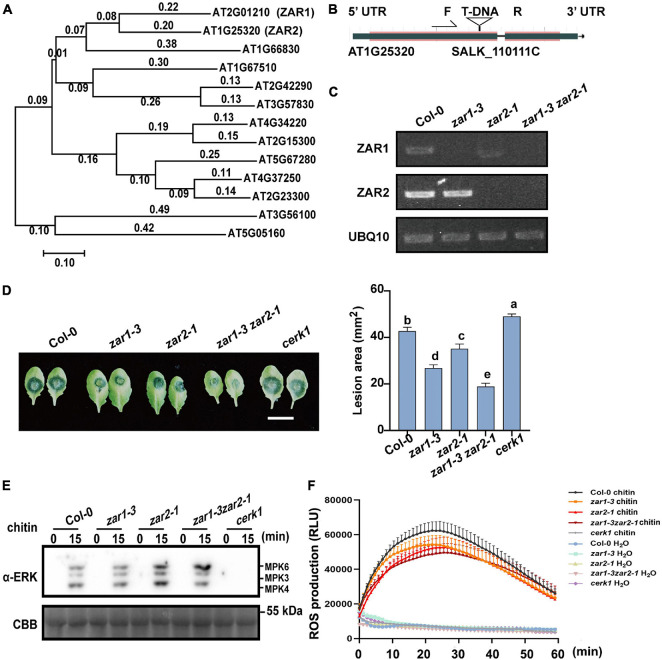
ZAR1 and ZAR2 function redundantly in defense against *B. cinerea.* **(A)** The unrooted phylogenetic tree was constructed based on the full-length amino acid sequences of *Arabidopsis* proteins using MEGA 7 software by the Neighbor-Joining method. AT1G25320 (ZAR2) is most homologous to ZAR1. Scale bar is indicated. **(B,C)** Identification of *zar1-3zar2-1* double mutant. *zar2-1* (SALK-110111c) is a T-DNA insertion mutant in the Col-0 ecotype. *zar1-3zar2-1* double mutant was obtained by crossing *zar1-3* mutant with *zar2-1* mutant. The transcription of *ZAR1* and *ZAR2* genes in this double mutant was examined by RT-PCR. *UBQ10* was used as an internal control. **(D)** Both ZAR1 and ZAR2 negatively contribute to resistance against *B. cinerea.* Lesions on the leaves of *zar1-3* mutant, *zar2-1* mutant, *cerk1* mutant, *zar1-3zar2-1* double mutant, and Col-0 plants were observed at 72 h after inoculation with *B. cinerea*. Lesion sizes were analyzed. Error bars represent SEM from means of 16 leaves from each line. Different letters indicate significant difference at *P* < 0.05, as determined by one-way ANOVA with Tukey’s post-test. Scale bar = 1 cm. **(E)** Chitin-triggered MAPK activation in *zar1-3* mutant, *zar2-1* mutant, *zar1-3zar2-1* double mutant, *cerk1* mutant, and wild type Col-0. 10-day-old seedlings were treated with 200 μg/mL chitin at the indicated time points. MAPK activation was detected with an anti-pERK antibody. *cerk1* mutant was included as a control. Equal loading is demonstrated by CBB (Coomassie brilliant blue) staining of Rubisco (below). The experiments were repeated three times, with similar results. **(F)** Leaves of the 4-week-old *zar1-3* mutant, *zar2-1* mutant, *zar1-3zar2-1* double mutant, *cerk1* mutant, and Col-0 plants were treated with 200 μg/mL chitin and incubated with luminol and horseradish peroxidase to detect ROS. Luminescence was recorded at different time points as indicated. Error bars represent SEM of data derived from replicate samples (*n* = 12). The chemiluminescence was measured in a time-course manner using a microplate reader (Tecan-Spark, Tecan).

Zygotic arrest 1 functions redundantly in zygotic division regulation (Yu et al., 2016). Although the *zar1-3* single mutant showed stronger *B. cinerea* resistance compared with Col-0, we hypothesized that there could be some functional redundancy of ZAR1 in plant defense. We performed phylogenetic analysis of ZAR1 homologs based on the full-length amino acid sequences obtained from the NCBI non-redundant protein database and identified a ZAR1-homologous kinase named ZAR2 (AT1G25320) (Figure 3A). We obtained the homozygous *zar1-3 zar2-1* double mutant by crossing *zar1-3* with *zar2-1* (Figures 3B,C). The *zar1-3 zar2-1* double mutant plants exhibited almost normal growth and development compared with single mutants, despite a minor reduction in the leaf size. After 72 h of *B. cinerea* infection, the average lesion size on Col-0 leaves was about 40 mm^2^, whereas the average lesion size on *zar1-3 zar2-1* double mutant leaves was about 20 mm^2^, much smaller than that of *zar1-3* single mutant, *zar2-1* single mutant, *cerk1*, and Col-0 plants (Figure 3D). This result indicated that ZAR1 and ZAR2 might function redundantly in defense against *B. cinerea* in *Arabidopsis*.

Since the negative role of ZAR1 in defense against *B. cinerea* seems to be independent of the chitin-triggered early PTI responses (Figures 2D,E), we further explored the chitin-triggered early responses in *zar1-3 zar2-1* double mutant plants. Notably, the chitin-triggered MAPK activation was slightly enhanced in *zar2-1* single mutant and *zar1-3 zar2-1* double mutant (Figure 3E). In addition, both *zar2-1* single mutant and *zar1-3 zar2-1* double mutant exhibited compromised ROS burst upon chitin treatment, similar to that of *zar1-3* plants (Figure 3F). These results indicated that ZAR1 and ZAR2 may play variable roles in chitin-triggered MAPK activation, as well as minor positive roles in ROS burst. Thus, the remarkably enhanced resistance against *B. cinerea* in *zar1-3 zar2-1* double mutant might be due to the potential regulatory pathways other than pattern triggered immunity (PTI) responses.

**FIGURE 4:**
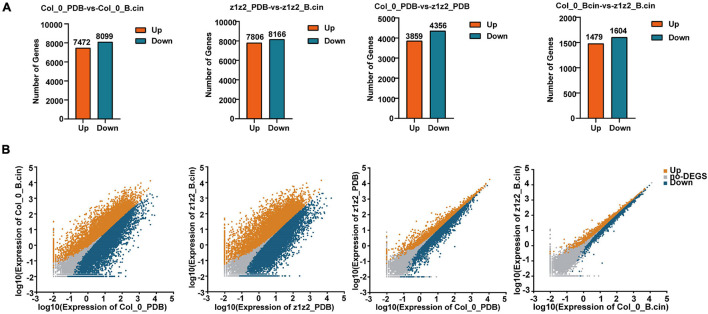
Data analysis of RNA-sequencing. **(A)** RNA-sequencing was performed in Col-0 and *zar1-3zar2-1* double mutant after 28 h of treatment with *B. cinerea* spray or PDB. Each treatment contained three biological replicates. Total RNAs derived from Col-0 or *zar1-3zar2-1* double mutant plants upon *B. cinerea* infection or PDB were compared between different genotypes or treatment conditions. Numbers of up- and down-regulated genes were illustrated (*P-*value < 0.05, fold change > 2). z1z2_PDB: *zar1-3zar2-1* double mutant plants treated with PDB; z1z2_B.cin: *zar1-3zar2-1* double mutant plants treated with *B. cinerea*; Col_0_PDB: Col-0 plants treated with PDB; Col_0_B.cin: Col-0 plants treated with *B. cinerea*. **(B)** The scatter plots illustrate differentially expressed genes between Col-0 and *zar1-3 zar2* double mutant. Genes up-regulated, down-regulated, or unchanged were shown in orange, blue, or gray, respectively. | log2FC| > 1 and FDR < 0.05 were set as a cut off.

**FIGURE 5:**
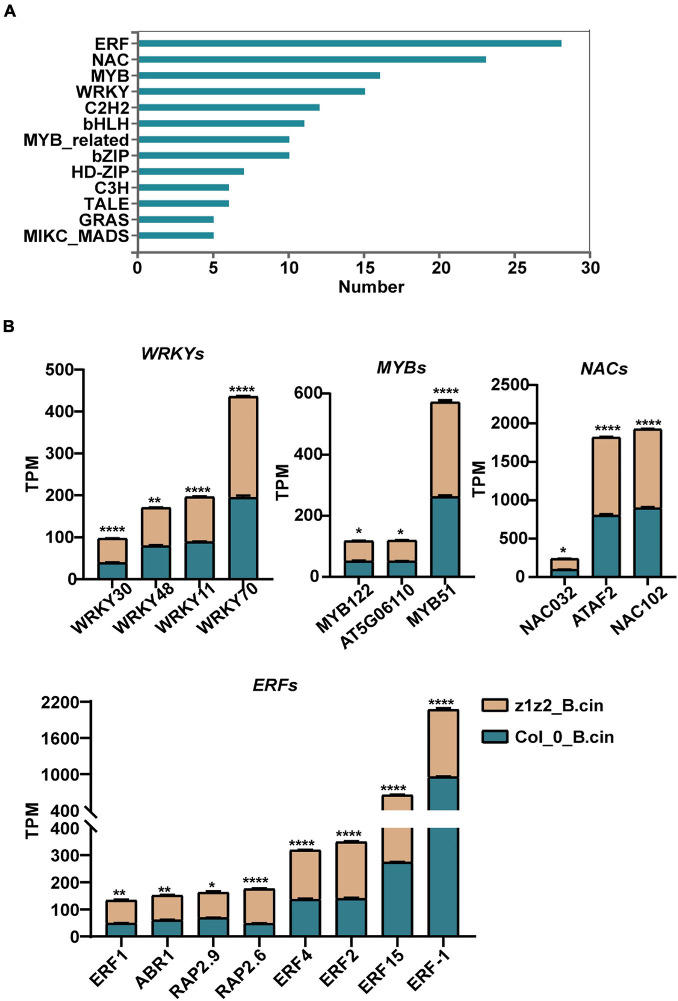
Gene Ontology (GO) analyses of differentially expressed transcription factor genes between *zar1-3zar2-1* and Col-0 plants upon *B. cinerea* infection. **(A)** Top 10 enriched clusters (GO analysis) of transcription factors genes based on data of the differentially expressed genes between Col-0 (*B. cinerea* treatment) and *zar1-3 zar2* (*B. cinerea* treatment) detected in the RNA-sequencing. **(B)** TPM (transcripts per Million) value of four defense-related transcription factor gene subgroups (*WRKYs*, *MYBs*, *NACs*, and *ERFs*) derived from the differentially expressed genes between Col-0 and *zar1-3zar2-1* mutant after *B. cinerea* infection. Asterisks indicate significant differences between *zar1-3zar2-1* mutant and Col-0 plant (*P < 0.05, **P < 0.01, ****P < 0.0001, Student’s *t*-test).

We further explored the downstream mechanism of ZAR1/ZAR2-related defense against *B. cinerea* using transcriptome analysis. We performed transcriptome sequencing in Col-0 and *zar1-3 zar2-1* double mutant plants 28h after treatment with *B. cinerea*. Total RNAs derived from Col-0 or *zar1-3 zar2-1* double mutant plants, treated with *B. cinerea* spore or PDB (as a mock treatment) spray, were compared between different genotypes and treatments. The numbers of up- and down-regulated genes (*P*-value < 0.05, fold change > 2) in the samples of 1) PDB-treated Col-0 compared with *B. cinerea*-treated Col-0 plants, 2) PDB-treated *zar1-3 zar2-1* compared with *B. cinerea*-treated *zar1-3 zar2-1* plants, 3) PDB-treated Col-0 compared with PDB-treated *zar1-3 zar2-1* plants, and 4) *B. cinerea*-treated Col-0 compared with *B. cinerea*-treated *zar1-3 zar2-1* plants, are illustrated in Figures 4A,B (Supplementary Table 1). Interestingly, there were many genes encoding transcription factors (TFs) among the DEGs between Col-0 and *zar1-3 zar2-1* after *B. cinerea* treatment. The top four enriched clusters analyzed through GO were defense-related transcription factors, including *ethylene response factors* (*ERFs*), *MYB domain proteins* (*MYB*s), *NAC domain containing proteins* (*NACs*), and *WRKY DNA-binding proteins* (*WRKYs*) (Figures 5A,B).

**FIGURE 6:**
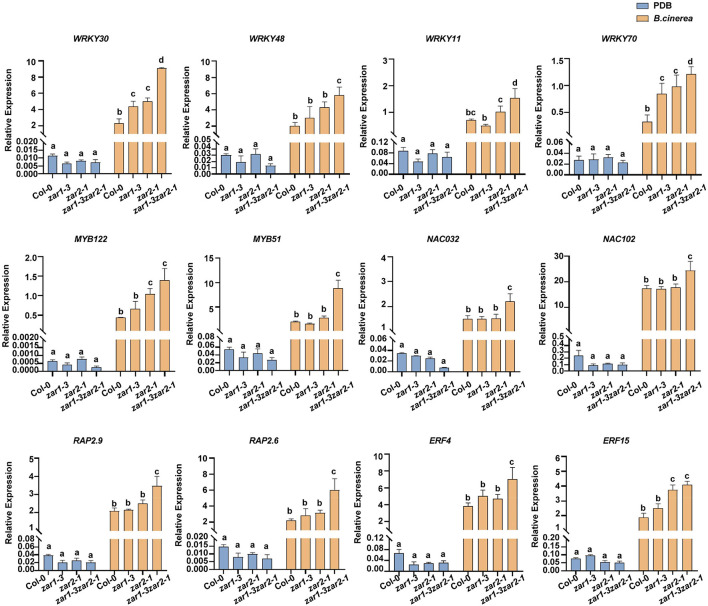
The relative expression levels of defense-related transcription factor genes induced by *B. cinerea* were enhanced in *zar1-3zar2-1* double mutant. The relative expression levels of defense-related transcription factor genes upon *B. cinerea* infection or PDB treatment were evaluated in *zar1-3* mutant, *zar2-1* mutant, *zar1-3zar2-1* double mutant, and Col-0 plant. Twelve-day-old seedlings were inoculated with *B. cinerea* spores or PDB for 28 h. The relative expression levels of defense-related TF genes were analyzed by qPCR, *UBQ5* was used as an internal control. The relative expression was calculated by the transcription level of each gene relative to *UBQ5* upon *B. cinerea* or PDB treatment. The different letter indicates significant difference (*p* < 0.05), as determined by one-way ANOVA with Tukey’s post-test.

The expression profiles of these TF genes were further validated with quantitative RT-PCR. In the absence of *B. cinerea* (PDB treatment), the basal transcriptional levels of these investigated genes were much lower than that of the *UBQ5* gene (Figure 6). After *B. cinerea* infection, the expression of *WRKY30*, *WRKY48*, *WRKY11*, and *WRKY70* was remarkably increased in *zar1-3 zar2-1*, compared with single mutants and Col-0 plants (Figure 6). In addition, the expression levels of *MYB122*, *MYB51*, *NAC032*, *NAC102*, as well as several *ERF* genes, *RAP2.9*, *RAP2.6*, *ERF4*, and *FER15*, were strongly up-regulated upon *B. cinerea* infection in the *zar1-3 zar2-1* double mutant. The relative expression level of these genes in *zar1-3 zar2-1* was much higher than those of Col-0 plants (Figure 6). Thus, upon *B. cinerea* infection, the transcript levels of multiple defense-related TF genes in the *zar1-3 zar2-1* double mutant were increased to much higher levels compared with those in single mutants and Col-0 plants, which was consistent with the remarkably enhanced resistance observed in the *zar1-3 zar2-1* double mutant.

## Discussion

Plants have evolved various cell-surface RLKs that perceive diverse signals, such as phytohormones and peptides derived from plants, and pattern molecules derived from microbes. The coordination of growth and defense that depends on such receptors improves the ability of plants to adapt to environmental changes. In our current study, we demonstrated that the zygotic division regulator ZAR1 could directly bind to the defense protein CERK1. Furthermore, we showed that this interaction depended on dephosphorylation at the Y428 residue of CERK1 (Figure 1). Moreover, ZAR1 functioned redundantly with its homolog ZAR2 in plant defense against *B. cinerea* (Figures 2–6). Our results suggested a possible functional connection between ZAR1 and CERK1, each of which was originally identified as being involved in separate processes. Notably, ZAR1 is an LRR-RLK (Yu et al., 2016). LRR-RLKs are critical proteins involved in processes of plant development and stress responses in *Arabidopsis*. For example, the bacterial MAMP cognate receptors FLS2 and EFR are LRR-RLKs that modulate plant immune signaling (Gomez-Gomez and Boller, 2000; Zipfel et al., 2006). Moreover, LRR-RLKs play multiple roles during plant growth and development. For example, the BRI1-associated receptor kinase 1 (BAK1) functions in both brassinosteroid (BR)-dependent growth regulation and plant innate immunity in *Arabidopsis* (Chinchilla et al., 2007; Heese et al., 2007; Kim et al., 2013). The LRR-RLK ERECTA recognizes epidermal patterning factors (EPFs)/EPF-like proteins (EPFLs) and is essential in plant growth and development (Torii et al., 1996; Shpak et al., 2003; Meng et al., 2012). ERECTA can also regulate immune responses in *Arabidopsis* (Godiard et al., 2003; Jorda et al., 2016). In the current study, our findings identified crosstalk between the LRR-RLK ZAR1 and the LysM-RLK CERK1, as well as revealed that ZAR1 has multiple roles, functioning in both zygotic division and pathogen defense.

ZYGOTIC ARREST 1 can interact physically with the heterotrimeric G-protein Gβ (AGB1) and calmodulin to regulate the zygote development (Yu et al., 2016). ZAR1 and AGB1 form a complex, and the kinase activity of ZAR1 is activated by the interaction of AGB1 (Yu et al., 2016). Interestingly, AGB1 has been reported to be a minor positive regulator of chitin-induced ROS burst (Liu et al., 2013). We similarly observed that loss of ZAR1 function impaired the chitin-induced ROS burst in the *zar1-3* mutant (Figure 2E). Thus, both ZAR1 and AGB1 may play minor roles in the positive regulation of chitin-triggered early responses. However, it was paradoxical that loss of function of ZAR1 homologs enhanced the plant defense against *B. cinerea*. We speculated that ZAR1 was involved in the regulation of defense against *B. cinerea* independent of the chitin- trigged PTI responses, although ZAR1 could be recruited by the chitin receptor complex depending on the dephosphorylation of the residue 428 tyrosine of CERK1. Moreover, the ambivalent effects of ZAR2 deficiency on chitin signaling in the *zar1-3 zar2-1* double mutant further implied that ZAR1 homologs contributed to *B. cinerea* resistance beyond chitin signaling.

**FIGURE 7:**
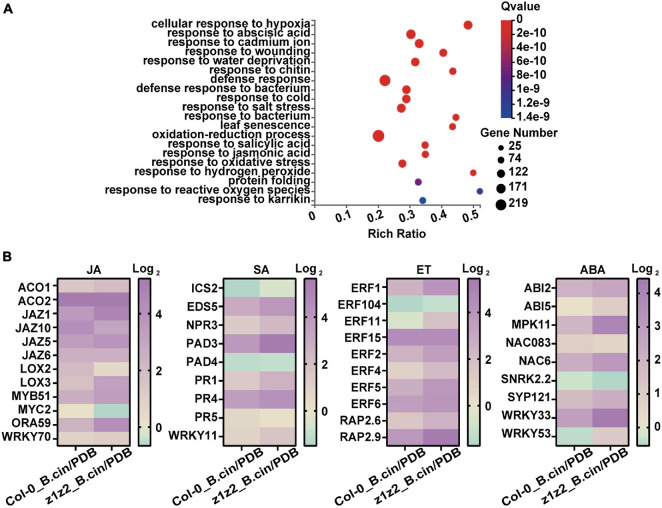
Analysis of differentially expressed genes involved in defense-related hormone pathways in *zar1-3 zar2-1* and Col-0 plants upon *B. cinerea* infection. **(A)** Top 20 GO enrichment of the genes detected in the transcriptomes that have significant differences in Col-0 plants (*B. cinerea* treatment) and *zar1-3zar2* plants (*B. cinerea* treatment). **(B)** Expression profiles of various JA, SA, ET, and ABA-related genes in Col-0 and *zar1-3zar2-1* double mutant compared between different treatment conditions (Col-0_ B.cin/PDB: Col-0 samples treated with *B. cinerea* compared with Col-0 samples treated with PDB, z1z2_B.cin/PDB: *zar1-3zar2-1* double mutant samples treated with *B. cinerea* compared with *zar1-3zar2-1* double mutant samples treated with PDB). Heatmap showed TPM levels of DEG genes from RNA-sequencing.

In host plants upon pathogen infection, defense signals driven by the plant immune responses are ultimately transmitted into the nucleus and the massive transcriptional reprogramming was governed by a complex gene regulatory network. TFs are key components in this regulatory cascade and function in plant resistance to pathogens by activating and repressing the expression of multiple genes. Upon *B. cinerea* infection in the *zar1-3 zar2-1* double mutant, the levels of induced transcription for various defense-related transcription factors were much higher than those of Col-0 plants, suggesting global activation of defense-related gene expression (Figures 5, 6). Furthermore, genes in pathways associated with defense-related hormones, such as the jasmonate (JA), salicylic acid (SA), ethylene (ET), and abscisic acid (ABA) pathway, were much more highly activated in the *zar1-3 zar2-1* double mutant compared with Col-0 plants upon *B. cinerea* infection (Figure 7), which still needs further validation.

In conclusion, our study established that ZAR1 played multifaceted functions in addition to zygotic division regulation in *Arabidopsis*. It was curious that loss of function of ZAR1 enhanced resistance against *B. cinerea*, independent of the intact chitin-induced MAPK activation and compromised chitin-elicited ROS burst in the *zar1-3* mutant. Our results highlighted the possibility that ZAR1 homologs could contribute to fungal resistance beyond chitin signaling. Recently, the important role of crosstalk between RLKs in regulating plant immunity has been recognized (Tang et al., 2017; Gong et al., 2019). Receptors can integrate their signaling to regulate defense responses (Macho and Zipfel, 2014; Tang et al., 2017; Miao et al., 2020). As LRR-RLKs, ZAR1 homologs could perceive ligand signals besides chitin and would have the potential to integrate differential signaling pathways to precisely regulate defense responses in plants upon pathogen infection, a concept that needs further investigation.

## Data Availability Statement

The datasets presented in this study can be found in online repositories. The names of the repository/repositories and accession number(s) can be found below: http://www.ncbi.nlm.nih.gov/bioproject/, PRJNA757130.

## Author Contributions

LC and BL designed the study and wrote the manuscript. LC, JX, YS, YL, JL, and HC performed the research. H-BW analyzed the data. All authors contributed to the article and approved the submitted version.

## Conflict of Interest

The authors declare that the research was conducted in the absence of any commercial or financial relationships that could be construed as a potential conflict of interest.

## Publisher’s Note

All claims expressed in this article are solely those of the authors and do not necessarily represent those of their affiliated organizations, or those of the publisher, the editors and the reviewers. Any product that may be evaluated in this article, or claim that may be made by its manufacturer, is not guaranteed or endorsed by the publisher.

## Footnotes

1
www.Arabidopsis.org.uk

2
http://geneontology.org/

3
http://www.ncbi.nlm.nih.gov/bioproject/757130

4
http://motif-x.med.harvard.edu/
