# Supplementary material for: The Zygotic Division Regulator ZAR1 Plays a Negative Role in Defense Against Botrytis cinerea in Arabidopsis
Source: Front Plant Sci. 2021 Oct 26;12:736560. doi: 10.3389/fpls.2021.736560 (PMC8575783; doi:10.3389/fpls.2021.736560)
Supplement: Supplementary file 2 [file Table_2.DOCX]

**Table S2 Oligonucleotide primers used in this study**

| **Primers** | **Sequences (5’-3’)** |
| --- | --- |
| ZAR1-RT-F | GGAATCGGAATTGTCTACAA |
| ZAR1-RT-R | CAGCGATGTTAGCTAACCGT |
| ZAR2-RT-F | CGGGAACGGGATTGTGTATA |
| ZAR2-RT-R | ATGTTCCAGCAATGCTAGAG |
| PUC-ZAR1-FLAG-SpeI-F | CTGGCGCGCCACTAGTATGTTGGCCTCGCTGATCAT |
| PUC-ZAR1-FLAG-SpeI-R | CGATGGATCCACTAGTATCGCCGGCCACGGGTAATC |
| WRKY30-qPCRPCR-F | AACAATGCCGATACAAAACCTC |
| WRKY30-qPCR-R | CGCATTTGAAGCATATAGGCAT |
| WRKY48-qPCR-F | TCCCCAATAACAACAACAACAC |
| WRKY48-qPCR-R | TTCTTCTTTGCCTTCAACTGTG |
| WRKY11-qPCR-F | CGCATCTCAGAAACTACAGAGT |
| WRKY11-qPCR-R | AATCGAGTGTTACACTAGACGG |
| WRKY70-qPCR-F | GAGTCGACTATACTTGAGGACG |
| WRKY70-qPCR-R | ACGTGTGGTTTCCTATGTATGT |
| MYB122-qPCR-F | GACCAAAAGCTTATCGCCTATG |
| MYB122-qPCR-R | AATCTTCCTCGTCTTGGCTAAA |
| MYB51-qPCR-F | ACACCAGTTTCATCGAACTTTG |
| MYB51-qPCR-R | GTTTTCAACACAAGACTCCTCC |
| NAC032-qPCR-F | GATTGGACGATTGGGTATTGTG |
| NAC032-qPCR-R | AACGGAAAATCAAGGGCATTAC |
| NAC102-qPCR-F | CTGATAAACCGATCGGAAAACC |
| NAC102-qPCR-R | CTCGTGCATAATCCAATTCGTT |
| RAP2.9-qPCR-F | AGCCTAATAAACGATCACGGAT |
| RAP2.9-qPCR-R | CCTCTTCAGGGAAATTGAGTCT |
| RAP2.6-qPCR-F | GTTGGAACTCAGACGATTCAAC |
| RAP2.6-qPCR-R | AGAGTTGGACATTGATCGATGT |
| ERF4-qPCR-F | CTTCTCCTCCGACGTTAGTTG |
| ERF4-qPCR-R | CAGGTCCAAAAAGTAGACAGGA |
| ERF15-qPCR-F | TCTTGCAACACTTAATTTCCCG |
| ERF15-qPCR-R | ACGAAGAAGAAGAGCAAGAAGA |
| UBQ10-F | GATCTTTGCCGGAAAACAATTGGAGGATGGT |
| UBQ10-R | CGACTTGTCATTAGAAAGAAAGAGATAACAGG |
| UBQ5-F | AATGTGAAGGCGAAGATCCAAGAC |
| UBQ5-R | AGACGGAGGACGAGATGAAGC |
